# The Effect of Ca Dopant on the Electrical and Dielectric Properties of BaTi_4_O_9_ Sintered Ceramics

**DOI:** 10.3390/ma14185375

**Published:** 2021-09-17

**Authors:** Asad Ali, Muhammad Hasnain Jameel, Sarir Uddin, Abid Zaman, Zafar Iqbal, Qeemat Gul, Fozia Sultana, Muhammad Mushtaq, Khaled Althubeiti, Rafi Ullah

**Affiliations:** 1 Department of Physics, Riphah International University, Islamabad 44000, Pakistan; zafar.iqbal@riphah.edu.pk; 2 Department of Physics, Government Post Graduate College Nowshera, Nowshera 2500, Pakistan; 3 Institute of Modern Physics, Northwest University, Xi’an 710069, China; mhasnainjamil@gmail.com; 4 Department of Physics, Government Degree College Hayatabad, Peshawer 2500, Pakistan; sariruddin@uop.edu.pk; 5 Department of Physics, University of Okara, Okara 56300, Pakistan; gulphysicist@gmail.com; 6 Department of Chemistry, University of Science and Technology China, Hefei 230026, China; fsultana@mail.ustc.edu.cn; 7 Faculty of Materials and Manufacturing, Beijing University of Technology, Beijing 100124, China; mushtaqphy009@yahoo.com (M.M.); Rafi_hassan2011@yahoo.com (R.U.); 8 Department of Chemistry, College of Science, Taif University, P.O. Box 11099, Taif 21944, Saudi Arabia; k.althubeiti@tu.edu.sa

**Keywords:** (Ba_1−x_Ca_x_)Ti_4_O_9_ ceramics, dielectric properties, complex impedance

## Abstract

The current research examines the impact of Ca^2+^ substitution on the phase and electrical properties of (Ba_1−x_Ca_x_)Ti_4_O_9_, (x = 0.0, 0.3, 0.6, and 0.9) sintered pellets synthesized by solid-state reaction method. The as-synthesized samples were analyzed using X-ray diffraction (XRD) and impedance spectroscopy. The emergence of orthorhombic phase fit into space group Pnmm was revealed by XRD, and the addition of Ca resulted in a considerable shift in grain size. Dielectric properties were determined using an impedance spectroscopy in a wide frequency range from 1MHz to 3 GHz. The dielectric properties i.e., dielectric constant (ε*_r_*) and dielectric loss (tanσ), were measured at 3 GHz frequency. The frequency-dependent parameters such as conductivity, dielectric constant, and dielectric loss indicated that the relaxation process is a Maxwell–Wagner type of interfacial polarization. The improved dielectric properties and low energy loss have made (Ba_1−x_Ca_x_)Ti_4_O_9_ a prominent energy storage material. This study provides the possibility to improve its dielectric properties and reduce energy loss, making it an excellent energy storage material.

## 1. Introduction

Owing to remarkable chemical as well as electrical properties, barium titanate (BT) has traditionally been regarded as developing dielectric material. Barium tetra titanate nanoparticles have recently received a lot of interest due to their numerous applications in modern communication technologies, such as software radio systems, global positioning systems (GPS), and environmental monitoring satellites. In addition, a high-quality resonators component has been used for high-speed communication systems, data storage devices, rechargeable batteries, and many more microwave dielectric applications. To improve the microwave dielectric properties, most laboratories and enterprises provide some micro-level oscillators and microwave frequency filters at low cost [1,2]. Ceramics compounds i.e., BaTi_4_O_9_, BaTi_5_O_11_ and Ba_2_Ti_9_O_20_, exhibit better relative permittivity values and insignificant dielectric loss in the region radio frequency [3]. Moreover, a complex perovskite AB_4_O_9_ type, i.e., Barium tetra titanate (BT_4_), where A and B sites usually represent cations and many researchers make additions to these sites, enhances the microwave dielectric properties [4,5,6,7]. Meanwhile, doping divalent metals (Ca^2+^, Sr^2+^, Sn^2+^) onto BaTi_4_O_9_ improves the dielectric properties (ε*_r_*) of the sample, such as the quality factor (Q) and temperature coefficient of resonant frequency (τ*_f_*), which are important in the manufacture of electronic devices. When Ca^2+^ occupies the position of barium in the BT4 sample, the orthorhombic phase of Ba_1−x_Ca_x_Ti4O_9_ (BCT_4_) emerges. In addition, the doping of Ca^2+^ improves the dielectric and electrical properties of the ceramic compounds for electrical and dielectric resonator applications [8]. Because of their good microwave dielectric properties, the majority of investigations have been focused on diverse dielectric ceramic materials, particularly ceramics based on BT and polymer-related substances. BaTi_4_O_9_ ceramics have good dielectric properties i.e., high relative permittivity (ε*_r_* = 36), good quality factor (Q = 3260 GHz), or low loss (Tanδ = 0.00048) and temperature coefficient of resonant frequency (τ*_f_* = +24 ppm/°C). BT4 or BT-based solid solutions with further B-site or A-site doping components (i.e., Ca^2+^, Sr^2+^, Zr^4+^, Sn^4+^) are the most important dielectric ceramics, which improve the microwave dielectric properties [9,10,11]. In addition, the microwave dielectric properties of electro-ceramics are heavily influenced by the production strategies and dopant content [12].

In the present research work, the phase, electrical, and dielectric properties of the sintered solid solution ceramics (Ba_1−x_Ca_x_)Ti_4_O_9_ (x = 0.0, 0.3, 0.6, and 0.9) has been investigated.

## 2. Experimental Method

The solid solution of (Ba_1−x_Ca_x_)Ti_4_O_9_ (x = 0.0, 0.3, 0.6,and 0.9) calcined ceramics was obtained using conventional route. High-grade reactant raw powders were selected and weighed according to the stoichiometric ratio; these powders were purchased from Aldrich Chemicals (BaCO_3_, 99.9% and CaCO_3_, 99.9%) and sigma (TiO_2_, 99.9%). These weighed powders were mixed in a distilled water medium for six (06) hours using horizontal ball milling. The wet mixture was dried by keeping it in oven at 100 °C for one day. The dried material was re-milled (in dry form) for two (2) hours before being calcined in an alumina crucible of high purity for 3 h in a furnace at 900 °C with 5 °C/min heating cooling rate. The calcined powder was manually crushed for one hour with a mortar and pestle to prevent agglomeration. The ground fine calcined powder was pressed into cylindrical dye with a diameter of 10 mm and thickness of 5 mm using of manual pellet press machine (CARVER, Washington D.C., USA) at 100 MPa pressure. These pellets were indorsed with sintering at 1000 °C for 4 h in air with a heating cooling rate of 5 °C/min. The Archimedes principle can be used to measure the apparent bulk densities of the ceramic samples. At microwave frequency, the samples’ dielectric characteristics and minimal dielectric loss were determined using an impedance analyzer (Agilent 4287A, Keysight, Santa Rosa, CA, USA).

## 3. Results and Discussions

### 3.1. Phase Analysis

**Figure 1:**
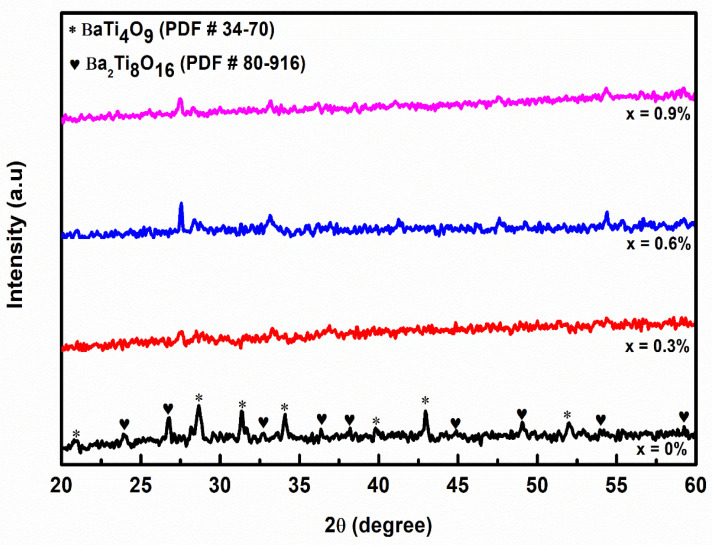
XRD pattern of (Ba_1−x_Ca_x_)Ti_4_O_9_ (x = 0.0, 0.3, 0.6 and 0.9) ceramics powders calcined at 900 °C in air [13].

The phase analysis profile of (Ba_1−x_Ca_x_)Ti_4_O_9_ with various Ca^2+^ contents is shown in Figure 1 [13]. The crystal structure (i.e., orthorhombic structure) along with space group Pnmm elementary assembly of BaTi_4_O_9_ (compared with reference card number 34–70) is responsible for all diffraction peaks, with lattice parameters of a = 6.294(5) Å, b = 14.532(11) Å, and c = 3.797(3) Å. Many peaks have been identified previously along with secondary phase (i.e., Ba_2_Ti_8_O_16_) with reference card number (PDF No.80–916) [13]. The influence of Ca^2+^ concentrations on the structural properties of (Ba_1−x_Ca_x_)Ti_4_O_9_ ceramics has been recorded [14]. It is worth noting that certain peaks vanish while others shift to lower 2θ values as the Ca^2+^ content in (Ba_1−x_Ca_x_)Ti_4_O_9_ rises. This could be attributed to micro strain, solid-state inhomogeneity, or the substitution of Ba^2+^(R_Ba_ = 1.44) for the comparatively enormous radius of Ca^2+^ (R_Ca_ = 1.57Å) for Ba^2+^ (R_Ba_ = 1.44Å) [15].

The crystalline nature of orthorhombic structure barium tetra titanate is attributed to the highly intense peaks and strong diffraction pattern as shown in Figure 1 (BaTi_4_O_9_), which increase with the increase of Ca^2+^ contents in (Ba_1−x_Ca_x_)Ti_4_O_9_ concentration, indicating that the particles gradually participate with the increase of Ca content.

The density (ρ_x_) of X-ray can be observed using the following formula:

(1) $$\rho_{x}=\frac{\mathrm{ZM}}{N_{A}}$$

The number, of atoms per unit cell is eight, where ‘ρ_x_’ is the calculated density, ‘M’ is the molecular weight of compound while ‘N_A_’ is the Avogadro number

(2) $$\rho_{x}=\frac{nM}{N_{A}V}$$

where ‘*n*’ is the no. of moles and ‘V’ is the volume.

Theoretical densities of the samples can be calculated using Equation (1) while experimental densities can be determined using the Archimedes principle (density meter MD-35). Then we find the relative densities as shown in Table 1.

### 3.2. Dielectric Characteristics

#### 3.2.1. Complex Dielectric Constant

The mobility of the electric dipole generated by the applied electric field is usually the source of dielectric relaxation. The impact of applied electric field on the dielectric materials has been described using the Debye–Scherer relaxation model [16]. The complex dielectric constant is calculated as follows:

(3) $${ɛ}^{*}={ɛ}^{\prime }-j\varepsilon^{\prime \prime }$$

where ‘ε*’ is complex permittivity, ‘ε′’ is real permittivity and ‘ε″’ is imaginary permittivity.

**Figure 2:**
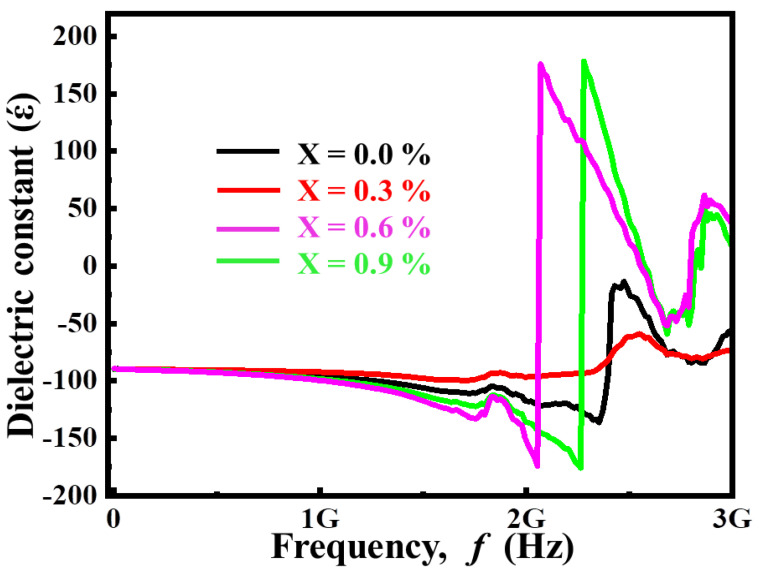
Variation of dielectric permittivity with frequency for (Ba_1−x_Ca_x_)Ti_4_O_9_ (x = 0.0, 0.3, 0.6 and 0.9) sintered ceramics at 1000 °C for 4 h.

A capacitor’s storage capacity may be increased using dielectric materials. Therefore, the material’s capacitance is proportional towards its dielectric constant.

(4) $$\varepsilon \text{′}=\frac{\mathrm{Cd}}{\varepsilon_{o}A}$$

where ‘C’ is capacitance, ‘d’ is thickness, ‘A’ is area and ‘ε_o_’ is the permittivity of free space. Figure 2 shows the variation of dielectric constant with frequency for (Ba_1−x_Ca_x_) Ti_4_O_9_ (x = 0.0, 0.3, 0.6, and 0.9) sintered ceramics, at 1000 °C for 4 h. At low frequencies, the dielectric constant is very large, but it drops dramatically as frequency increases, eventually becoming constant at high frequencies. Ionic, electronic, interface, and orientation polarization are the four types of polarization that can be used to understand this behavior. Because each of these polarization mechanisms has its unique relaxation time, changes in applied frequency have an impact on charge carrier hopping. [17,18]. At low applied frequencies, all polarization mechanisms contribute, but as the frequency rises, some polarization mechanisms (such as dipole polarization and interface polarization) are unable to rotate in the external electric field. As a result, the overall contribution of all mechanisms tends to reduce, and the values of dielectric constant drop with increasing frequency, becoming independent at higher frequency, as shown in Figure 2 i.e., the dielectric constant (*εʹ*) increases with increasing Ca^2+^ concentration until x = 0.9, where it starts to decline. Because the atomic polarizability of Ca^2+^ is higher than that of Lanthanum (La), the dielectric constant increases until x = 0.9. Though, at contents (x = 0.9) the sample porosity increases and, the grains size decreases, leading to an increase in resistivity, and polarization becomes incredibly difficult, potentially lowering the dielectric constant. The largest value of actual dielectric constant is attained for compositions with larger grain size [19]. According to this study, charge carrier mobility promotes dipolar orientation.

When the parallel plate capacitor is connected to an external power supply source, the current leads the voltage by phase angle 90° which causes the power dissipation due to the leakage of current. As a result, the tangent loss may be determined using the following formula:

(5) $$\tan\delta =\frac{1}{2\pi fC_{P}R_{P}}$$

where ‘2πf’ is the angular frequency, ‘C_p_’ is parallel capacitance, ‘R_p_’ parallel resistance while ‘tan δ’ is loss tangent or dielectric loss.

**Figure 3:**
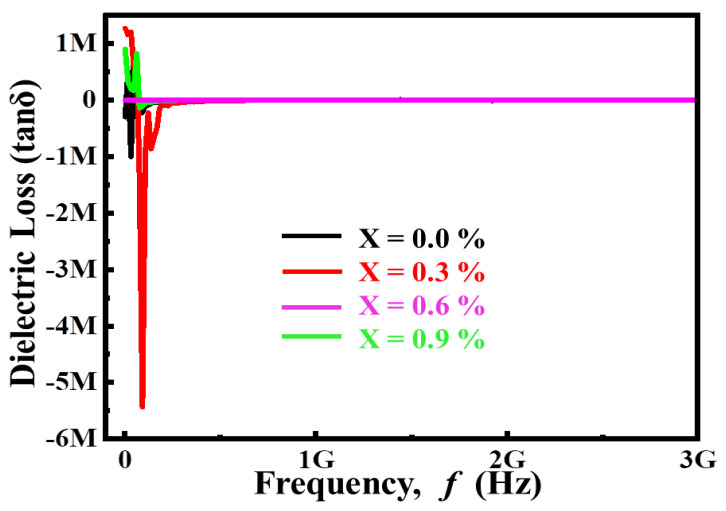
Variation of dielectric loss as a function frequency for (Ba_1−x_Ca_x_)Ti_4_O_9_ (x = 0.0, 0.3, 0.6 and 0.9) sintered ceramics at 1000 °C for 4 h.

The ideal capacitor has zero loss angle, and consumes zero power. Power dissipations are commonly referred to as dielectric loss in commercial capacitors and will be evaluated. This is depicted in Figure 3, where the variation in dielectric loss (tan δ) is a function of frequency (f) for ceramic pellets manufactured from (Ba_1−x_Ca_x_) Ti_4_O_9_ (x = 0.0, 0.3, 0.6, 0.9) sintered at 1000 °C for 4 h. It is noted that both have the same tan loss trend relative to the Ca^2+^ content. These trends follow the Maxwell–Wagner interface polarization model, which aligns with Koop’s Phenomenological Theory (KPT). The dielectric materials are modeled as multilayer capacitors with grain edges and grains [19,20]. The grain boundary with deficient conductivity is more efficient in the low-frequency range because of internal morphological defects, whereas the smooth grain is more active at high frequency. The relaxation peaks for distinct components arise at different frequencies, as shown in Figure 3. According to polarization resonance, each polarization mechanism has its own relaxation frequency, which causes resonance when the relaxation frequency matches the applied frequency. As a result, the presence of various component peaks is due to the relaxation phenomenon of these samples. With a rise in Ca content, the relaxation peak shifts towards low frequency, implying that the relaxation time may increase. The decrease in peak intensity with increasing substitution content could be attributable to a reduction in defects and contaminants.

#### 3.2.2. Conductivity Analysis

Conductivity is defined as the movement of charge carrier under the action of applied field. The conductivity increases gradually at lower frequencies, but at higher frequencies the conductivity abruptly increased. At lower frequencies, the resistive nature of grain boundaries becomes more complicated, resulting in a low conductivity value being observed [21]. The high conductive structure of grains becomes highly active at higher frequencies, resulting in increased charge carrier hopping between the ions and increased conductivity [22]. The net conductivity of ceramics can be found using Jonsher’s Power Law (JPL).

(6) $$\sigma_{\mathrm{total}}=\sigma_{\mathrm{dc}}-{A\omega }^{s}$$

where ‘σ_dc_’ is the conductivity of DC, ‘A’ is the pre-exponential factor and ‘s’ is the exponent. The phrase ‘ac conductivity’ encompasses the entire concept of Aω^s^. The following formula [23] can be used to calculate ac conductivity:

(7) $$\sigma_{\mathrm{ac}}=\varepsilon \text{′}\varepsilon_{o}\omega \tan\delta$$

where (ω = 2πf) is Angular frequency.

**Figure 4:**
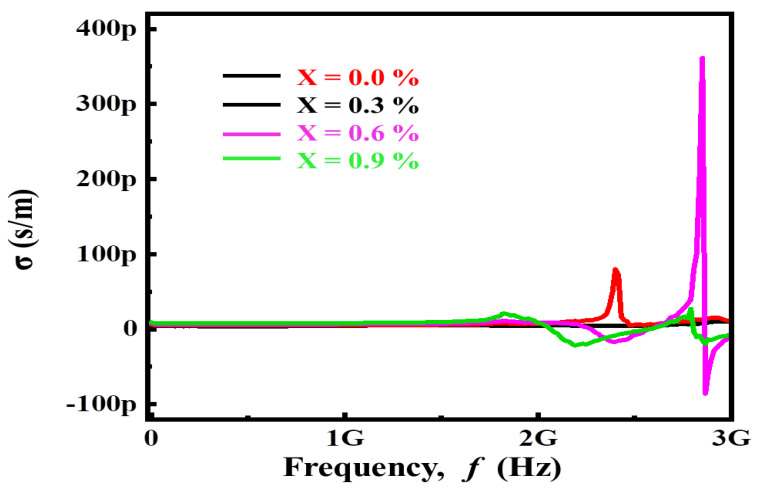
Variation of conductivity as a function frequency for (Ba_1−x_Ca_x_)Ti_4_O_9_ (x = 0.0, 0.3, 0.6 and 0.9) sintered ceramics.

Figure 4 displays a variation of conductivity (σ_ac_) versus frequency (f) for (Ba_1−x_Ca_x_) Ti_4_O_9_ (x = 0.0, 0.3, 0.6, and 0.9) sintered ceramic pellets at 1000 °C for 4 h. The change in conductivity is minimal at first, but as the frequency increases, the conductivity increases dramatically. The hopping process and carrier mobility may be stimulated by increasing frequency; however, the free carrier may also consider the bound charge to conduct at high frequency. The cationic disorder caused by Ca^2+^ substitutions of smaller cations at the A-site may boost the conductivity by creating oxygen vacancies in the lattice skeleton at the 48f site [24].

#### 3.2.3. Quality Factor

Ca^2+^ concentration has a significant impact on microwave dielectric parameters such as dielectric constant (ε_r_), quality factor (Q × f) and temperature coefficient of resonant frequency (τ*_f_*). At a frequency of 3 GHz, the dielectric characteristics of sintered ceramics samples were investigated.

**Figure 5:**
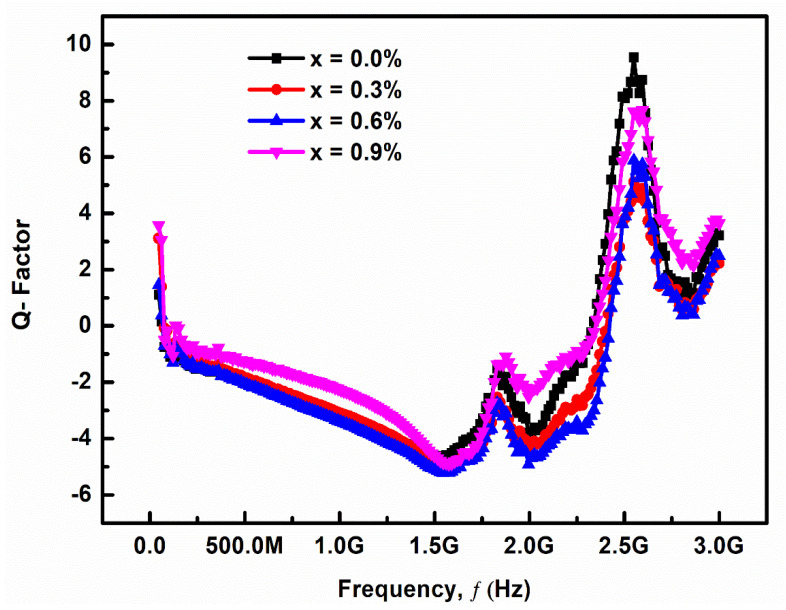
Variation Q factor with frequency for (Ba_1−x_Ca_x_)Ti_4_O_9_ (x = 0.0, 0.3, 0.6 and 0.9) sintered ceramics at 1000 °C for 4 h.

Figure 5 shows the variation of Q factor with frequency (f) for (Ba_1−x_Ca_x_) Ti_4_O_9_ (x = 0.0, 0.3, 0.6, and 0.9) sintered ceramic at 1000 °C for 4 h. At 1.5 GHz, there is a good quality factor and low loss dielectrics, making it excellent for microwave dielectric resonator applications. The temperature coefficient of resonance frequency (τ*_f_*) is virtually zero for good tunable and silent circuits. However, we observed that due to the formation of the secondary phase, the values of temperature coefficient of resonance frequency (τ*_f_*) increases with increasing the Ca^2+^ concentrations. As the sintering temperature rises, porosity decreases and grains become more tightly connected, increasing density. Similarly, sintering temperature has an impact on relative permittivity.

#### 3.2.4. Complex Impedance Analysis

Impedance spectroscopy is a useful tool for investigating the role of grains and grain boundaries, as well as the polarization mechanism. Some properties of solids depend on the capacitance and resistance values of individual microstructures, which are crucial in determining the dielectric response of materials. This technique enables us to estimate the resistance and capacitance provided by the bulk and grain boundaries and helps us find the relaxation time and frequency. The frequency dependence of impedance in complex form can be written as:

(8) $$Z^{*}=Z\prime +\mathrm{jZ}\prime \prime$$

Dielectric loss

(9) $$\tan\delta =\frac{\varepsilon \prime \prime }{\varepsilon \prime }=\frac{Z\prime }{Z\prime \prime }$$

Complex impedance

(10) $$Z^{*}=\frac{\varepsilon \prime \prime }{C_{o}\omega (\varepsilon^{\prime^{2}}+\varepsilon^{{\prime \prime }^{2}})}+j\frac{-\varepsilon \prime }{C_{o}\omega \left(\varepsilon^{\prime^{2}}+\varepsilon^{{\prime \prime }^{2}}\right)}$$

where ‘Z*’ is complex impedance, ‘Z*′*’ is real impedance while ‘Z″’ is imaginary impedance.

**Figure 6:**
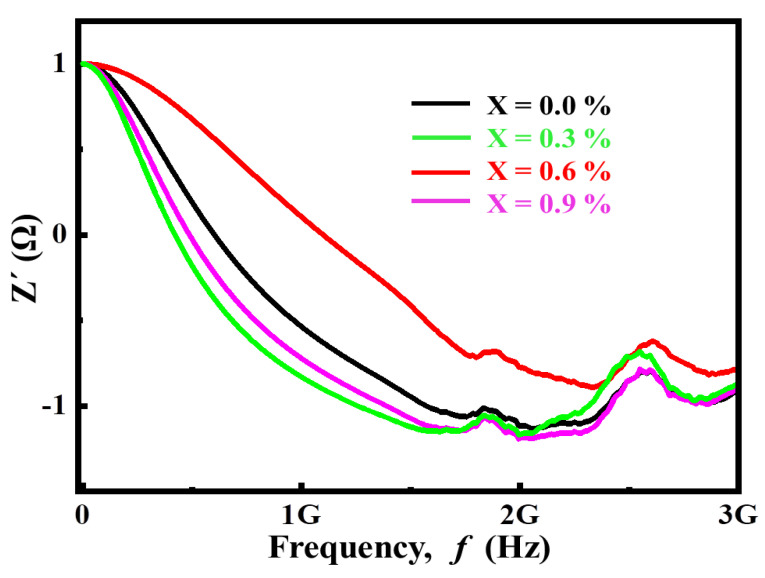
Frequency-dependent variation of real part of impedance for (Ba_1−x_Ca_x_)Ti_4_O_9_ (x = 0.0, 0.3, 0.6 and 0.9) sintered ceramics at 1000 °C for 4 h.

**Figure 7:**
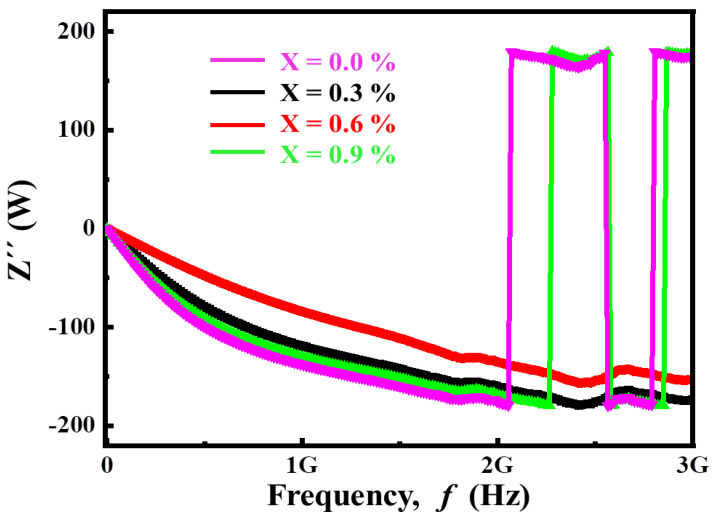
Frequency-dependent variation of imaginary parts of impedance for (Ba_1−x_Ca_x_)Ti_4_O_9_ (x = 0.0, 0.3, 0.6 and 0.9) sintered ceramics at 1000 °C for 4 h.

Figure 6 demonstrates the frequency dependence of real impedance, which shows a diminishing trend as frequency increases. It can be observed that the impedance is very high in the low-frequency range, demonstrating the benefit of the grain boundary. Due to the effect of conductive grains, it decreases with the increase of frequency. In complex impedance analysis, the plot between the real part of impedance Z*′* and the imaginary part of impedance Z″ is known as the Nyquist plot. The analysis shows that the magnitude of Z*′* decreases with increasing frequency and temperature, indicating the ac conductivity $(\sigma_{\mathrm{ac}}$) of the sample increases. The increase of conductivity can be explained as the reason for oxygen deficiency distribution at high temperature. At low frequency, Z*′* decreases significantly with the increase of Ca^2+^ substitution. At high frequency, the value of Z*′* seems to be frequency independent, which indicates that the increase of Ca^2+^ concentration increases the temperature and leads to the increase of conductivity. The merging of the Z*′* curves in the higher frequency region is probably the space charge released by the decrease of the barrier performance of the samples. It is also observed that the frequency at which the Z*′* curves coincide increases with the increase of Ca^2+^ content. On the other hand, Figure 7 shows that Z″ shifts towards higher value with the increase of frequency.

#### 3.2.5. Complex Modulus Analysis

To analyze the electrical and relaxation properties of materials, complex impedance spectroscopy is an important technique. It provides important information about the stability of electrical behavior by studying the hopping of charge carriers and material homogeneous or heterogeneous properties. The electrical properties of bulk samples can be obtained by this technique because the study is more sensitive to the bulk properties of samples. The complex modulus M* is the inverse of complex dielectric constant ε*, i.e., $M^{*}={\left(\varepsilon^{*}\right)}^{-1}={\left(\varepsilon \prime -j\varepsilon \text{″}\right)}^{-1}=M\prime +\mathrm{jM}\prime \prime$. Where M*′* and M″ are the real and imaginary parts of the complex modulus, respectively, as determined by the following relations [25]:

Complex electrical Modulus

(11) $$M^{*}=M\prime +jM\prime \prime$$

(12) $$M^{*}=\frac{\varepsilon \prime }{\varepsilon \prime^{2}+\varepsilon {\prime \prime }^{2}}+j\frac{\varepsilon \prime \prime }{\varepsilon \prime^{2}+\varepsilon {\prime \prime }^{2}}$$

where ‘M*’ complex modulus, ‘M*′*’ real modulus while ‘M″’ is imaginary modulus.

**Figure 8:**
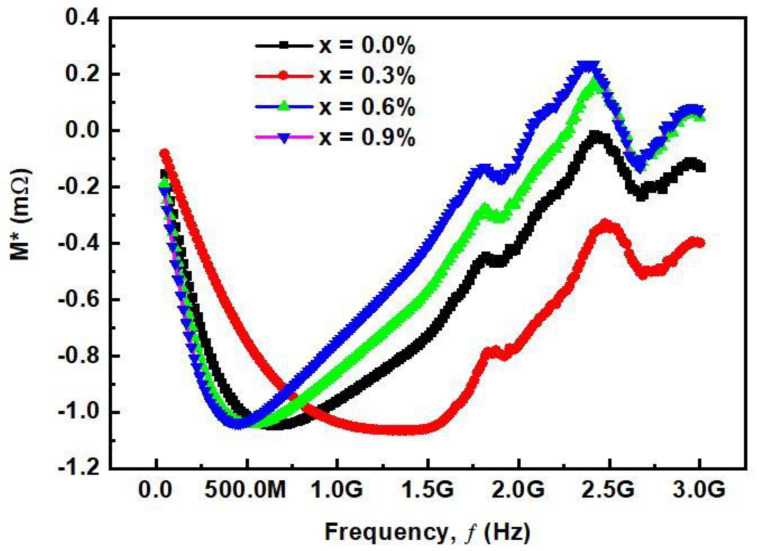
Frequency-dependent variation of complex modulus for (Ba_1−x_Ca_x_)Ti_4_O_9_ (x = 0.0, 0.3, 0.6 and 0.9) sintered ceramics at 1000 °C for 4 h.

Figure 8 shows the frequency dependence of complex modulus M*. M* increases as the frequency increases from low to high. This trend can be characterized by the asymmetric nature, and relates to the short-range mobility of ions and electrons. Two types of behavior can be seen here: first, a decreasing trend at lower frequencies, and then an increasing trend at higher frequencies. Second, the peak value first moves to high frequencies until x = 0.9 and then again moves to low frequencies. The low-frequency region shows the long-distance charge mobility, while the high frequency region shows the short distance mobility due to the limitation of the potential well. The peak shift describes the increase and then decrease in the relaxation process, which is almost consistent with the dielectric properties. Therefore, it can be concluded that the contribution of grains and grain boundaries exists in the samples. These results show that the increase of Ca^2+^ substitution enhances the grain boundaries, which shift the plots towards the high value of M′, leading to an increase in capacitance of the samples.

## 4. Conclusions

The solid-state solution of (Ba_1−x_Ca_x_)Ti_4_O_9_ (x = 0.0, 0.3, 0.6, and 0.9) ceramics has been investigated. The phase analysis shows that the impurity-free orthorhombic phase structure along with space group (Pnmm) is formed at (x = 0.0), the lattice parameters decrease with the substitution of Ca^2+^, and the unit cell volume also decreases. Impedance analysis revealed that the value of dielectric constant increases while increasing the Ca^2+^ contents due to their high atomic polarizability and decreases further due to increasing the sample porosities. Frequency-dependent ac conductivity was found to increase with increasing Ca^2+^ content due to the growth of grain boundaries and ionic polarizabilities. Complex modulus analysis investigated the various types of charge carrier mobility. The obtained results from this research are suitable for charge storage devices as well as for microwave wireless communication system applications.

## Figures and Tables

**Table 1:** Sintered (Ba_1−x_Ca_x_)Ti_4_O_9_ ceramic compositions and their microwave dielectric properties.

X	Calcination Temperature	Sintering Temperature	ρ_exp_ (g/cm^3^)	ρ_x_ (g/cm^3^)	ρ_re_ (%)	ε_r_	Q×f (at 3 GHz)	Tan(δ)
0	900 °C/3 h	1000 °C/4 h	4.402	4.71	74.4	36.62	5669.75	0.00017
0.3	900 °C/3 h	1000 °C/4 h	4.593	5.95	76.28	37.88	6595.05	0.00015
0.6	900 °C/3 h	1000 °C/4 h	4.433	3.95	76.65	38.13	6732.67	0.00014
0.9	900 °C/3 h	1000 °C/4 h	4.691	4.23	77.85	77.85	58.6139	0.00017

X = contents, ρ_exp_ = experimental density, ρ_x_ = calculated density, ρ_re_ = relative density, ε_r_ = dielectric constant, Q × f = quality factor, Tan(δ) = loss tangent or dielectric loss.

## Data Availability

Generated data should be publicly available and cited in accordance with journal guidelines.
